# Do Esports Players Experience Pain? Pain Prevalence of Esports Players: a Systematic Review and Meta-analysis

**DOI:** 10.1186/s40798-025-00971-1

**Published:** 2026-01-03

**Authors:** Markus Soffner, Alexander Schmidt, Fabian Tomschi, Thomas Hilberg

**Affiliations:** Department of Sports Medicine, University of Wuppertal, Moritzstr. 14, D-42117 Wuppertal, Germany

**Keywords:** Pain prevalence, Esports players, Health, Gaming, Digital games, Musculoskeletal pain

## Abstract

**Background:**

Esports, defined as competitive video gaming, has grown significantly in popularity, drawing global audiences comparable to traditional sports. However, the sedentary and repetitive nature of esports activities raises concerns about musculoskeletal health. While some studies have examined pain prevalence in esports players, no comprehensive synthesis exists. This systematic review and meta-analysis investigated the overall pain prevalence of esports players, as well as its occurrence in different body regions.

**Methods:**

Following PRISMA guidelines, studies reporting pain prevalence in esports players were identified via PubMed, Web of Science, and Google Scholar. Random-effects meta-analyses were used to calculate one-year and seven-day prevalence rates, as well as pain by body region. Meta-regressions explored potential moderators.

**Results:**

Thirteen studies met the inclusion criteria, with six (553 participants) contributing to the meta-analysis. One-year pain prevalence was 73% ([95% CI: 0.58–0.89], I² = 61%, k = 2), while the seven-day prevalence was 44% ([95% CI: 0.38–0.49], I² = 0%, k = 3). The spine was the most affected region (41%, [95% CI: 0.26, 0.55], I² = 96%, k = 11), with neck pain being particularly prevalent (48%, [95% CI: 0.26, 0.70], I² = 94%, k = 4). Upper extremities were also frequently affected (31%, [95% CI: 0.18, 0.44], I² = 96%, k = 12), with the wrists being notably affected by pain (37%, [95% CI: 0.09, 0.66], I² = 97%, k = 4). Meta-regression suggested higher pain prevalence among mobile players and Asian participants compared to computer players and European participants, though these findings are exploratory due to the limited number of studies.

**Conclusions:**

In this meta-analysis a considerable prevalence of pain among esports players was observed, especially in the spine and upper extremities. However, the results should be interpreted with caution due to methodological heterogeneity and limited study numbers. Nevertheless, they emphasize the need for preventive strategies, such as regular breaks during prolonged sitting, ergonomic interventions and load management programs to optimize training volume and recovery, thereby reducing the risk of musculoskeletal strain. Future research should distinguish between acute and chronic pain, use standardized tools, and explore device-specific pain patterns to inform targeted interventions.

*Registration number* PROSPERO ID: CRD42024599339.

**Supplementary Information:**

The online version contains supplementary material available at 10.1186/s40798-025-00971-1.

## Key Points

- 73% of esports players reported pain in the past year, with the spine (41%) and upper extremities (31%) being most affected.
- Neck and wrist pain were particularly prevalent among esports players, highlighting key risk areas.
- Future studies should use standardized assessments, differentiate between acute and chronic pain and examine device-specific risk factors.

**Supplementary Information:**

The online version contains supplementary material available at 10.1186/s40798-025-00971-1.

## Background

 In recent years, interest in video gaming has continued to grow, making it one of the most popular leisure activities globally, with over 3.4 billion players worldwide [1]. Esports, defined as competitive video gaming, has given the industry an even greater boost. Meanwhile, professional competitions fill entire stadiums and attract millions of viewers via internet streams. For example, the numbers of spectators at international esports tournaments, such as the League of Legends World Championship or The International, played in the game Defense of the Ancients 2, are now comparable to the viewership of some traditional sports [2].

Although esports offers many opportunities for gamers, including financial rewards, global recognition, and career growth [3], the intense training characterized by long periods of sitting in monotonous postures and repetitive upper extremity movements associated with professional gaming have raised concerns about physical health [4–6]. This sedentary activity with repetitive, monotonous movements may be associated with an increasing risk of musculoskeletal disorders [7–9]. This can also lead to various issues, including wrist tendinitis, carpal tunnel syndrome, or musculoskeletal pain [10, 11]. When playing video games, the back, neck or upper limbs are most frequently affected by pain in most of the studies [8]. Furthermore, it was shown that playing video games for more than three hours a day increased the likelihood of musculoskeletal pain, suggesting that prolonged exposure to postures associated with gaming may exacerbate these health issues [8].

Existing individual studies on pain in esports players provide a first yet fragmented view of these health issues. Some studies report high rates of musculoskeletal pain, particularly in the hands, wrist, and lower back [12], while others suggest lower prevalence rates [13]. The specific causes of pain in esports are likely multifactorial, involving a combination of biomechanical, psychological, and lifestyle factors, such as prolonged screen and sitting time, stress, and inadequate physical activity [10]. Despite these insights, there is no consensus on the overall prevalence of pain among esports players, making it difficult to assess the extent of the pain burden or develop appropriate interventions for prevention. Furthermore, factors that moderate the relationship between competitive esports gaming and pain remain underexplored.

Given the growing popularity of esports and the increasing recognition of pain as a significant concern in this population, it is essential to synthesize existing data on pain prevalence. While individual studies are limited by small sample sizes, a more comprehensive meta-analytical picture of pain and its prevalence in esports players is lacking. Yet, this knowledge is essential for establishing recommendations for injury prevention, player health, and performance optimization. By providing a clearer understanding of the extent of pain in esports players, this work seeks to contribute to the development of preventive strategies and clinical recommendations for esports players. Furthermore, it will highlight gaps in the current literature and suggest directions for future research.

This systematic review and meta-analysis aims to estimate the prevalence of pain in esports players by synthesizing data from existing studies. Specifically, the focus is on (1) the overall prevalence rates of pain across different prevalence periods (i.e., one-year, seven-day) and (2) identifying the most commonly affected body regions within these different prevalence periods. In addition, (3) moderators that can have an influence on the prevalence of pain in esports players are identified.

## Methods

### Identification and Selection of Studies

A systematic review and meta-analysis of the existing scientific literature were conducted based on the guidelines recommended by the Preferred Reporting Items for Systematic Reviews and Meta-Analysis (PRISMA) statement [14]. The study selection process, methodological quality assessment, and data analysis were performed by two independent investigators (MS, AS). Discrepancies between the reviewers were settled through discussion with a third reviewer (FT). This meta-analysis was prospectively registered on PROSPERO on 20th of October 2024 (ID: CRD42024599339).

From 1st of November to 20 of December 2024, the online databases PubMed (Medline), Web of Science, and Google Scholar were used to search for relevant articles. The search covered all records from each database's inception to the final search date (20 December 2024). The search strategy was developed on the basis of the following PICOS selection criteria [15]: Population (P): Healthy male and female esports players or competitive players of any age or ethnicity without the presence or medical diagnosis of any chronic disease (e.g., cardiovascular, respiratory, neurological, endocrine, mental, metabolic, or cancer); Intervention (I): not applicable; Control (C): not applicable; Outcome (O): Point or periodic prevalence of pain (i.e., one-year, seven-day) in different body regions; Study design (S): Cross-sectional studies and cross-over or parallel group designs with data on pain prevalence at baseline. For interventional studies, only baseline data of pain prevalence were considered. Only studies that explicitly mentioned that they examined esports players or competitive players were included, as esports only encompasses competitive play. Due to the lack of standardized terminology in the studies included, all esports players and competitive gamers were grouped together and hereinafter referred to as “esports players”. Studies with casual gamers or exercised-based esports (e.g., Wii or virtual reality exergames) were excluded, as the majority of popular competitive esports games, such as League of Legends or Counter-Strike, are typically played in a sedentary setting. This restriction also helped reduce heterogeneity within the target group. Reviews and meta-analyses were also excluded from the analysis, but were examined for additional potentially relevant literature, as were the reference lists of studies considered suitable according to the inclusion criteria. Only studies in English were considered. In addition, studies were only included if they explicitly reported about pain. Studies reporting injuries or neurological symptoms such as numbness or tingling, for example, were not included when outcomes were reported in a combined or non-differentiated manner that did not allow the extraction of pain-specific data. Studies were also excluded if the outcome definition was ambiguous, for example when questions combined different constructs such as pain and strain (e.g., “Chronic Pain - Do you have any pain or strain” [16]). In such cases, it was not possible to determine whether affirmative responses referred to pain or other complaints, and therefore the data could not be reliably extracted. Furthermore, a specific period of prevalence had to be defined, such as pain at the current time, within the last seven days, over the past four weeks, or other time periods. This indicates that individuals experienced pain at least once during the specified period. Due to lack of data, it was not possible to differentiate between acute and chronic pain.

The detailed search strategy and the corresponding search strings for each database are provided in Supplementary Table S1, S2 and S3 in the supplementary information. Briefly, a combination of the primary terms “pain” and “esports” and their associated synonyms (e.g., musculoskeletal pain, physical symptoms for pain and videogaming, gaming for esports) was used for a comprehensive and systematic screening of the current literature.

### Screening Process and Extraction of Data

**Fig. 1:**
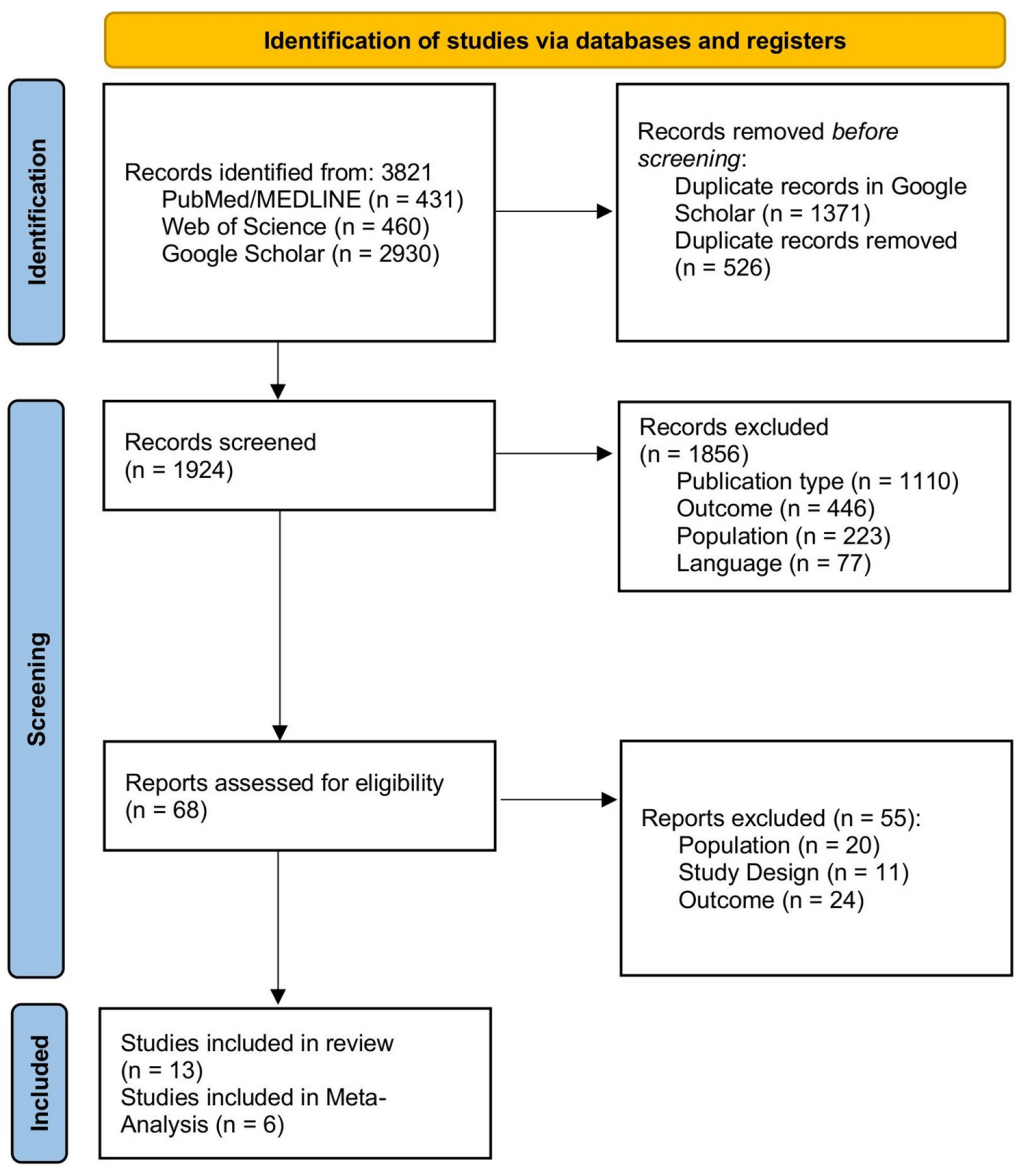
PRISMA diagram of the study screening process for examining the prevalence of pain in esports players

A preliminary literature screening and eligibility assessment based on titles and abstracts was conducted independently by two researchers (MS & AS). Subsequently, the remaining articles underwent full-text screening independently by the same two researchers, after which they were imported into Microsoft Excel (Microsoft Corporation, 2018, Redmond, Washington) for data management. The study selection process is outlined in Fig. 1. Any disagreements between the initial reviewers were resolved through consultation with an additional researcher (FT), leading to a consensus.

Once the included studies were determined, key study information was extracted independently by two researchers (MS & AS), including year of publication, country, study design, population, number of participants, age, sex, type of video gaming device, evaluation methods, prevalence period, prevalence rate in different body regions, overall prevalence rate and pain intensity. These data were added to the Excel sheet for data management. An overview of this information can be found in Table 1.

**Table 1:** Overview of the included studies including characteristics of participants, type of video gaming device, evaluation method, prevalence period and assessed body regions

Study, country	Study design	Population	Participants (males) age [years] mean ± SD	Type of video gaming device	Evaluation methods	Prevalence period	Pain in body regions (prevalence rate %)	Overall prevalence rate	Pain intensity (0–10)
Bahrilli et al. [26] (2022), Turkey	Cross-sectional survey	Professional and semi- professional athletes	47 (46) 20.98 ± 1.39	Computer, more n. a.	Non-standardized questionnaire	n. a.	Low back (42.5), upper back (36.1), neck (29.7), headache (23.4), hand-wrist (4.2)	n. a.	3.17 ± 1.81
DiFrancisco-Donoghue et al. [32] (2019), USA	Cross-sectional survey	College esports players	56 (n. a.) 18–22 n. a.	Computer, more n. a.	Non-standardized questionnaire	n. a.	Back or neck (41), wrist (36), hand (30)	n. a.	n. a.
Ekefjärd et al. [30] (2024), Sweden	Cross-sectional survey	Professional gamers	40 (n. a.) 18–22 (47. %), 23–27 (32.5%), 28+ (20%)	n. a.	NMQ	3 months	Headache (40.0), eyes (32.5), wrists (12.5), low back (7.5), shoulders (7.5), elbows (5.0), hands (5.0), fingers (2.5), neck (0.0)	62.5	n. a.
Gaasedal et al. [31] (2023), Denmark	Cross-sectional survey	Esports players	164 (153) 20.2 ± 4.2	n. a.	Non-standardized questionnaire	14 days	Back (50.0), neck and shoulder (36.0)	n. a.	n. a.
Hansen et al. [24] (2024), Denmark	Cross-sectional survey	Esports players	76 (66) 19.1 ± 2.9	Computer	Non-standardized questionnaire	7 days	Back (32.0), hand/fingers/wrist (24.0)	48.7	4.4 (95% CI 3.5–5.1)
Khan and Chaikumarn [25] (2024), Pakistan	Cross-sectional survey	Esports players	145 (111) 23.03 ± 2.64	Mobile	NMQ	7 days, 1 year	Neck (41.4), shoulders (31.0), low back (29.0), upper back (18.6), wrists (18.0), elbows (12.4), knees (11.7), hips/thighs (11.0), ankle/feet (9.7)^a^	n. a.	n. a.
Kurniawan et al. [13] (2024), Indonesia	Cross-sectional survey	Professional gamers	43 (42) < 25 (36) ≥ 25 (7)	Mobile	NMQ	1 year	Shoulder (30.2) hand (27.9), neck (23.2), wrist (16.3), low back (4.7), elbow (4.7)	65.1	n. a.
Lam et al. [27] (2022), China	Cross-sectional survey	Professional gamers	50 (50) 20.0 ± 1.67	Mobile	Non-standardized questionnaire	6 months	Neck (40.0), finger (38.0), headache (32.0), upper back (24.0), low back (20.0), eye (18.0), shoulder (16.0), wrist (8.0), knee (6.0)	n. a.	n. a.
Lindberg et al. [23] (2020), Denmark	Cross-sectional survey	Esports players	188 (184) 17.1 ± 2.3	Computer	Non-standardized questionnaire	7 days	Back (31.3), neck (11.3), shoulders (11.3), head (8.8), forearm (7.5), wrist (6.3), hand/fingers (5.), knee (5.0), thigh (3.8), ankle (3.8), shinbone (1.3), upper arm (1.3), chest (1.3), hip/groin (1.3), foot/toes (1.3), stomach (0.0), elbow (0.0)	42.6	4.0 ± 1.8
Mariano et al. [28] (2024), Philippines/ Japan	Cross-sectional survey	Competitive players	87 (79) 14–17 (18) 18–21 (42) 22–26 (27)	Computer, mobile, console	Non-standardized questionnaire	7 days, 6 months	Back (17.2), neck (17.2), wrist (17.2), shoulder (12.6), hand (11.4)^b^	n. a.	n. a.
Monma et al. [29] (2024), Japan	Cross-sectional survey	Esports players	79 (79) 21.6 ± 5.6	Computer, mobile, console	Non-standardized questionnaire	Point prevalence	Headache (30.4), neck (29.1), low back (29.1), wrist (6.3), finger (3.8)	n. a.	n. a.
Seng et al. [12] (2021), Malaysia	Cross-sectional survey	Esports players	69 (59) ≤ 21 (9) > 21 (60)	n. a.	BOSS	1 year	Hand/Wrist (79.7), neck (76.8), low back (75.4), upper back (66.7) shoulder (62.3), elbow (50.7), lower arm (44.9), thigh (21.7), ankle/foot (15.9), lower leg (14.5), knee (11.6)	n. a.	n. a.
Tholl et al. [22] (2024), Germany	Repeated measures design	Esports players	32 (32) 23.8 ± 3.4	Computer	NMQ (German)	7 days, 4 weeks, 1 year	Neck (50.0), upper back (31.3), low back (31.3), hands/wrists (28.1), shoulders (21.9), feet/ankles (15.6), elbows/forearms (12.5), knee (12.5), lower leg (12.5), Hip/thigh (9.4)^a^	37.5 56.25 81.25	n. a.

BOSS = Body Symptom Survey; n. a. = not accessible; NMQ = Nordic Musculoskeletal Questionnaire; ^a^ = prevalence of pain in last year; ^b^ = time period not clear (some studies reported multiple timeframes, but it was unclear which was presented)

The primary outcome of this meta-analysis was to determine the overall prevalence of pain as well as the prevalence across various body regions in esports players. The overall prevalence was defined as the presence of at least one painful site within the specified time period. All prevalence periods were considered to facilitate potential subgroup analyses. These data were extracted when available. In some articles, data were reported exclusively as absolute frequencies. These data were normalized relative to the total sample and converted into uniform prevalence estimates. If data were missing, the reviewers contacted the corresponding author to request the information. If no response was received within two weeks, a follow-up request was sent. Studies for which data remained unavailable after this process were excluded from the meta-analysis but retained for the systematic review.

### Risk of Bias Assessment

Two reviewers (MS & AS) evaluated the methodological quality of the included studies using the Risk of Bias Tool by Hoy et al. (2012) [17], which was specially developed for studies on prevalence. In contrast to tools for intervention studies (e.g., the Cochrane Risk of Bias Tool), this instrument addresses bias sources typical of prevalence studies, including sample representativeness, sampling techniques, case definition, and non-response bias. The tool consists of ten items plus a summary assessment for evaluating the overall risk of bias. The items can be divided into four domains: selection and non-response bias (external validity) as well as measurement bias and bias related to the analysis (internal validity). The external validity consists of four items: target population, sampling frame, random selection, and non-response bias. The internal validity consists of six items: data collecting from subjects, case definition, reliability and validity of the study instrument, same mode of data collection, length of the shortest prevalence period, and whether the numerator and denominator for the parameter of interest were appropriate. Each item is answered with either yes (low risk) or no (high risk), which applies to the study. For each item, corresponding examples are provided for assessing the respective item. The only exception is the summarizing assessment, which evaluates the overall risk of bias and is assessed as low, moderate, or high risk, independently of the other items. Due to the absence of standardized criteria for determining the classification of overall risk of bias, this item was disregarded because of its arbitrary categorization. During the development of the tool, this item demonstrated the highest levels of discrepancies [17]. These discrepancies arose, on the one hand, from the inability to accurately assess how individual items should be weighted and where exactly to draw the boundaries between the three levels of classification. On the other hand, this item uniquely involves three levels of assessment rather than two, which further complicates its application. Given the lack of clear and reproducible boundaries, the item was removed from the analysis.

### Statistical Analyses

The statistical software R was utilized for all analyses and the creation of figures. The main outcome was the prevalence of pain assessed over two temporal scales: a one-year period and a seven-day period. For both outcomes, random-effects meta-analyses were conducted using the R packages *meta* and *metafor*. Results of the meta-analyses were primarily clustered based on anatomical region, considering that the majority of studies reported pain prevalence in relation to specific body parts, while only a small subset provided information on overall pain prevalence. Anatomical regions were grouped into three aggregated rubrics:

Lower extremities: knee, lower leg, ankle/feet.

Spine: neck, upper back, lower back.

Upper extremities: shoulder, elbow, hand/wrist/fingers.

Prevalence data of hands, wrists, and fingers were combined into a single anatomical region category, i.e. hand/wrist/fingers. Similarly, data for the ankle and the feet were aggregated into the category ankle/feet. This approach was adopted due to inconsistencies in the presentation of pain prevalence across the individual body parts. When data on prevalence were reported separately for both body sites within one category, these values were pooled and subsequently used in the meta-analysis.

Inconsistency was assessed by quantifying between-study heterogeneity using the I² statistic. I² was interpreted as follows: 0% < I² < 40% as unimportant heterogeneity, 30% < I² < 60% as moderate heterogeneity, 50% < I² < 90% as substantial heterogeneity, and 75% < I² < 100% as considerable heterogeneity [18]. Another integral part of any well conducted meta-analysis is the evaluation of publication bias, which refers to the bias to disproportionally publish results with statistical significance [19]. However, considering that observational studies reporting data on prevalence are inherently less prone to publication bias as outcome calculation is not based on hypothesis testing and no universally accepted method for meta-analysis exists [20], publication bias was not evaluated in this study.

Meta-regression analyses were performed to identify determinants of the heterogeneity between studies [21]. For this purpose, the variables *continent*, *gaming modality*, *anatomical region*, and *risk of bias profile* were selected as potential moderators. The variable continent was limited to the Asian and European population, as studies conducted in other geographical regions were either unavailable or deemed ineligible for inclusion in the meta-analytical process. Initially, we intended to analyze a broader range of potential moderating variables (e.g., time spent playing video games or life-long gaming experience). However, this was not feasible due to data not being clearly formulated or not reported at all or dispersion of data across studies was too narrow (e.g., age, sex). Given the limited number of eligible studies, the meta-regression analyses should be considered exploratory in nature.

Given the limited number of studies, the meta-regression employed is thought to be more exploratory. The distribution of raw outcomes was substantially right-skewed and data were therefore log10-transformed, resulting in a distribution approximating normality. As prevalence rates were zero or approximated a value of zero in some cases, the continuity correction was applied for calculation of the standard error. The formula of the continuity correction used was as follows:

$$\:\text{P}\text{r}\text{e}\text{v}\text{a}\text{l}\text{e}\text{n}\text{c}\text{e}\:\text{a}\text{d}\text{j}\text{u}\text{s}\text{t}\text{e}\text{d}\:=\:\frac{(Event\:+\:0.5)}{(n\:+\:1)}$$

In this context, “event” refers to the occurrence of pain. The significance level was universally set at *p* < 0.05.

## Results

### Study Characteristics

In total, 13 of the 3821 screened studies met the inclusion criteria and were therefore included in the review. Due to the heterogeneous methodology, particularly with regard to the different prevalence periods, only six studies [12, 13, 22–25] with an overall of 553 participants could be included in the meta-analysis. An overview of the selection process is presented in Fig. 1.

Table 1 shows the study characteristics of the 13 included studies. The majority of the studies (seven) were carried out in Asia [12, 13, 25–29], five in Europe [22–24, 30, 31], and one in the USA [32]. With exception of one study, all studies were designed as cross-sectional surveys. Tholl et al. (2024) was a repeated measures design, but the participants had to complete a questionnaire in which, among other variables, musculoskeletal pain was recorded at baseline [22]. Overall, 1076 participants were involved. In eleven of the thirteen studies, the majority were male (83.7%); the other two studies did not record sex [30, 32]. The age of the participants was documented differently in the studies. Eight studies provided the mean value with standard deviation, from which an average age of 20.7 years was calculated [22–27, 29, 31]. The other studies either specified different age ranges or reported the proportion of individuals above or below a certain age. Seven studies [12, 22–25, 29, 31] described their population as esports players, one as competitive players [28], one as college esports player [32], one as semi- and professional players [26], and three as professional esports players [13, 27, 30]. Three studies included only mobile esports players [13, 25, 27], while three others considered only computer players [22–24]. Two studies included console, computer, and mobile players [28, 29] and two did not specify whether they were only computer players [26, 32]. In three studies, the gaming device was not specified [12, 30, 31]. The Nordic Musculoskeletal Questionnaire (NMQ) was the most frequently used tool for assessing pain and was applied in four studies [13, 22, 25, 30]. One study [12] used the Body Symptom Survey (BOSS), while the remaining eight studies used an unvalidated, self-designed questionnaire. The most frequently investigated prevalence period comprised a seven-day time frame and was used in five studies [22–25, 28]. Four studies investigated a prevalence period of one year [12, 13, 22, 25]. One study assessed point prevalence [29], two studies examined six-months prevalence [27, 28], and one study each investigated three-month [30], four-week [22] and 14-day prevalence periods [31]. Regarding the recorded anatomical regions where esports players experience pain, there was considerable heterogeneity, making it challenging to synthesize. Some studies assessed only anatomical regions [24, 31], whereas others surveyed up to 17 [23]. Furthermore, certain studies grouped anatomical regions together (e.g., back), while others assessed them individually (e.g., lower back, upper back), or did not mention specific regions at all (e.g., upper back). No study provided a precise differentiation between acute and chronic pain. Table 1 provides an overview of all anatomical regions recorded across the individual studies.

Only five studies reported an overall prevalence of pain among the esports players in their studies [13, 22–24, 30]. The intensity of the pain was only reported in three studies [23, 24, 26] and was therefore not considered in the meta-analytic process. The lowest average pain intensity was 3.2 ± 1.8 [26] and the highest was 4.4 (95% CI: 3.5–5.1) [24]. Due to heterogeneity and insufficient data, no subgroup-analyses based on playing time could be conducted. In the study by DiFrancisco-Donoghue et al. (2019), participants reported the highest gaming durations, ranging from 5.5 to 10.0 h per day, while Khan & Chaikumarn (2024) recorded an average gaming time of 8.14 ± 3.95 h per day [25, 32]. The lowest playing time was approximately 3 h per day and had been observed in several studies [22–24]. The years since the participants had started playing video games were approximately between 4 ± 2.5 [25] – 12 ± 4.3 years [22].

Hansen et al. (2024) and Lindberg et al. (2020) were the only studies that described pain in more detail with frequency and whether it was an activity-limiting factor [23, 24]. Accordingly, 16.5% and 6.25% perceived pain as an activity limiting factor. In addition, 54.0% and 42.5% respectively stated that the pain was experienced daily or several times a week.

### Results of meta-analysis

Several studies could not be included in the meta-analysis, because the pain prevalence period was not explicitly specified [26, 28, 32]. Another study [28] was excluded from the meta-analysis as two different prevalence periods were recorded, but the results could not be unequivocally assigned. In addition, there were only sufficient data for a meta-analysis regarding one-year and seven-day prevalence. Therefore, all other prevalence periods were also not included.

**Fig. 2:**
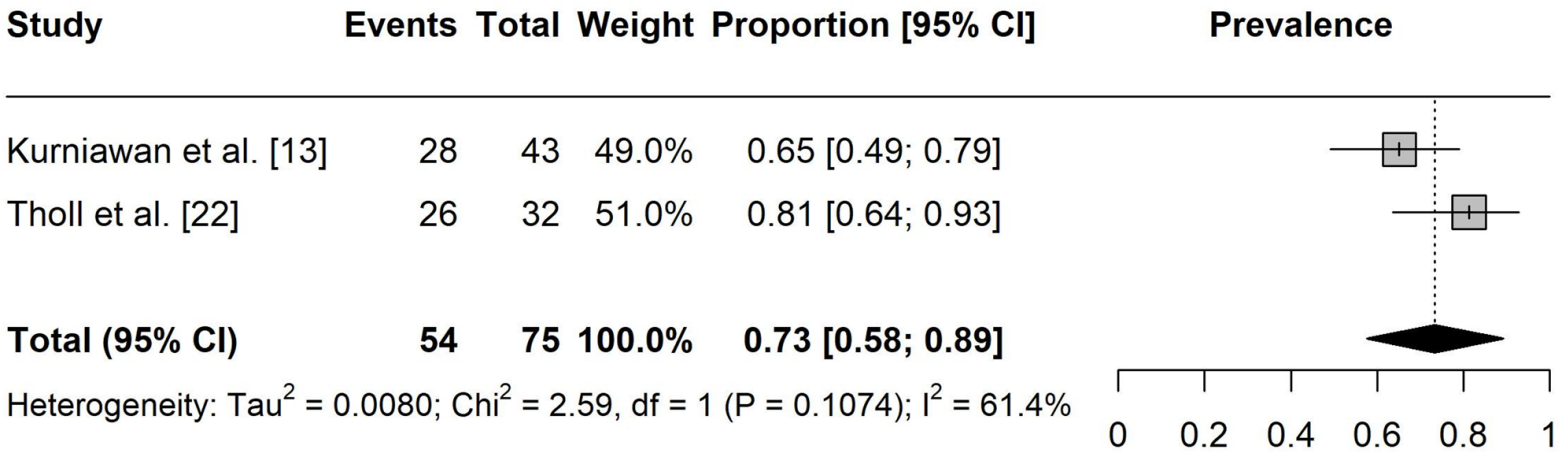
Forest plot of the overall one-year pain prevalence. Events = number of participants experiencing pain

**Fig. 3:**
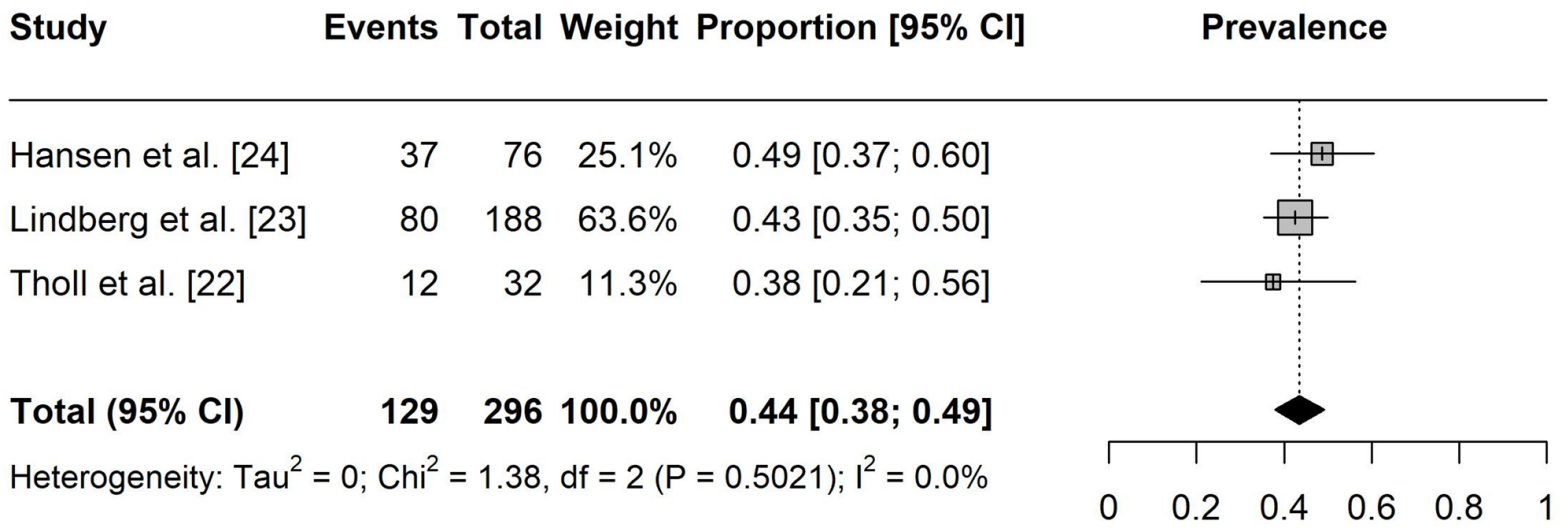
Forest plot of the overall seven-day pain prevalence. Events = number of participants experiencing pain

The one-year prevalence of pain among esports players was estimated at 73% ([95% CI: 0.58, 0.89], I² = 61%, k = 2) and the seven-day prevalence was 44% ([95% CI: 0.38, 0.49], I² = 0%, k = 3), as presented in Figs. 2 and 3.

**Fig. 4:**
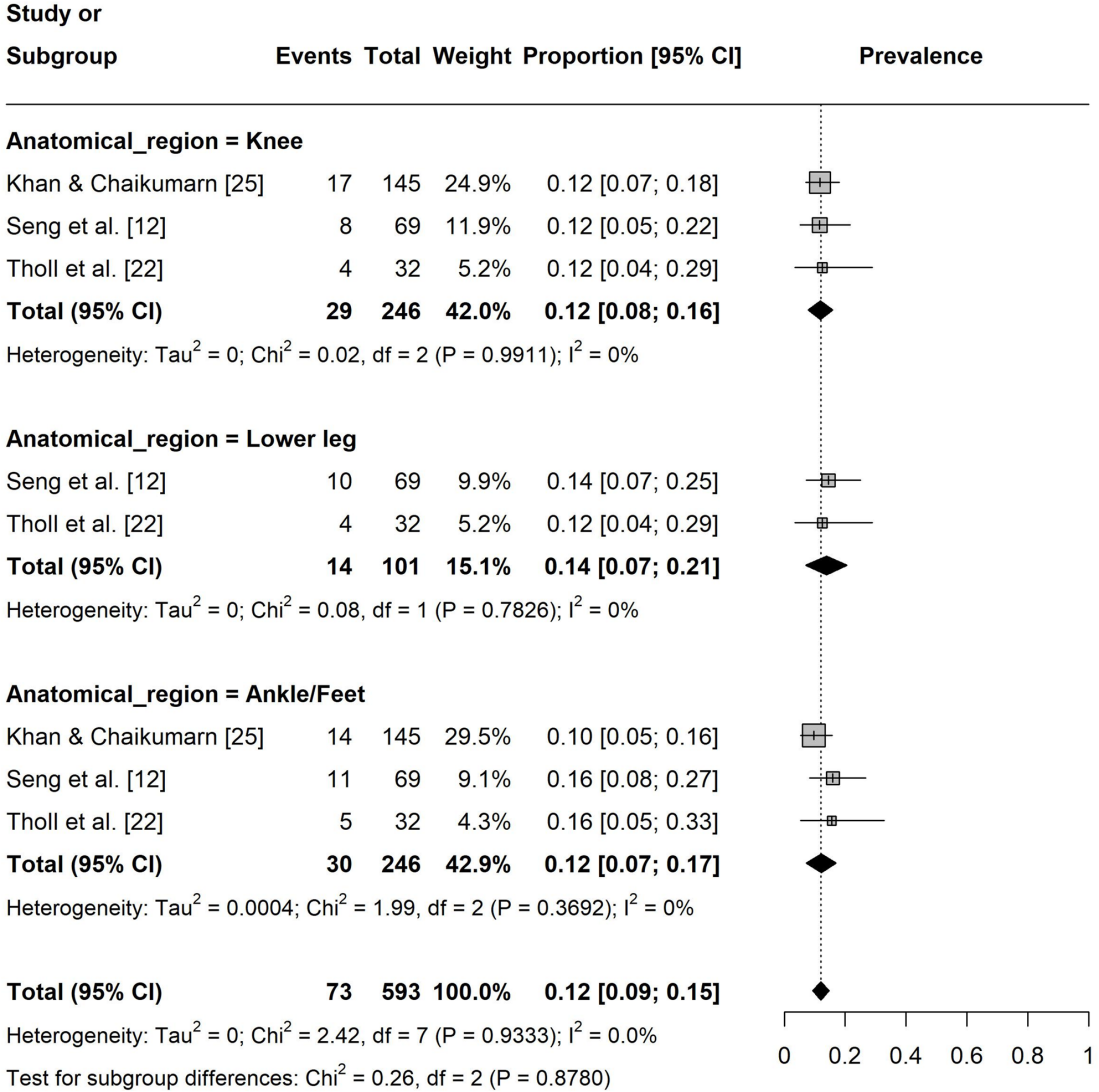
Forest plot of the one-year pain prevalence in the lower extremities. Events = number of participants experiencing pain

**Fig. 5:**
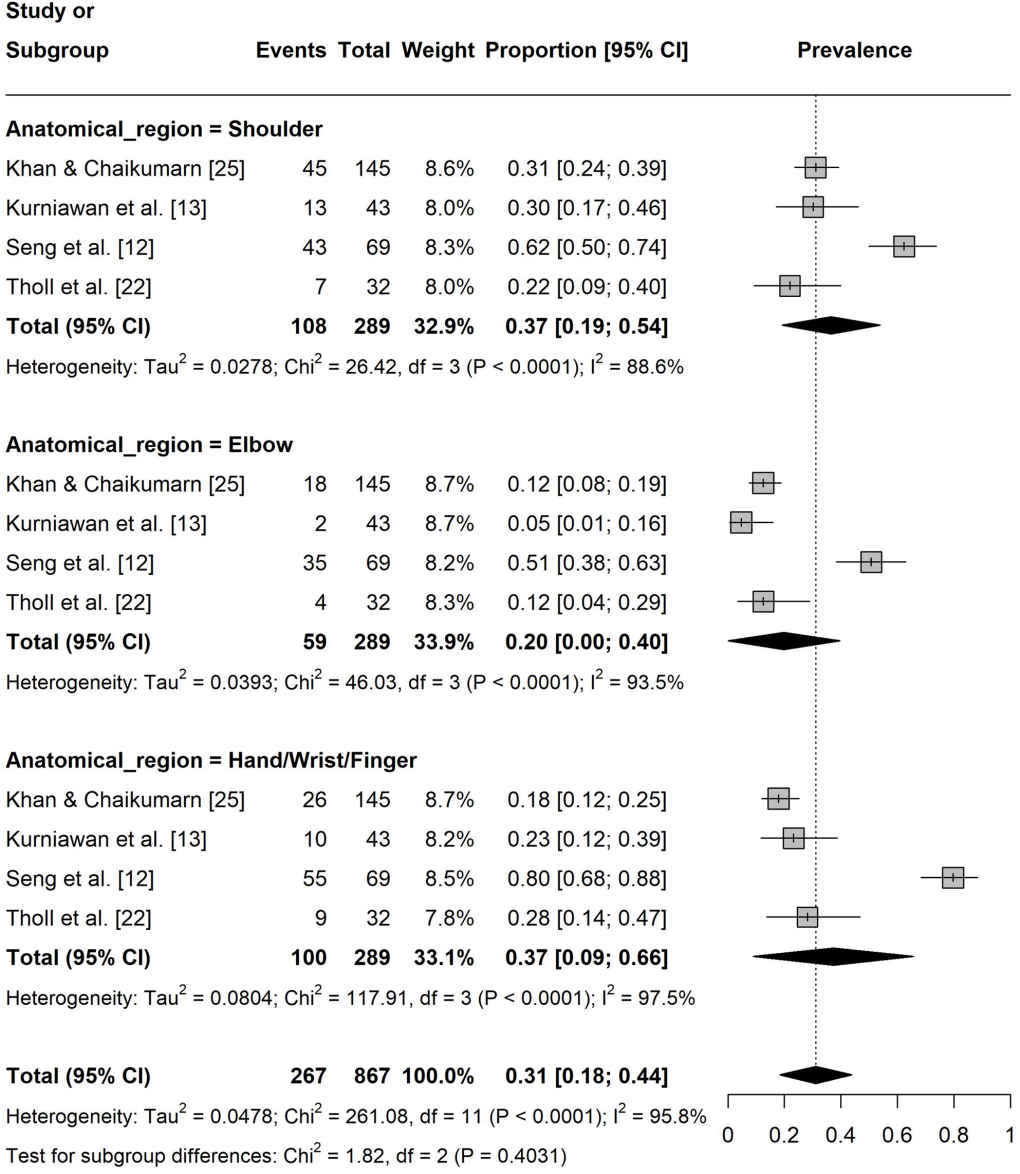
Forest plot of the one-year pain prevalence in the upper extremities. Events = number of participants experiencing pain

**Fig. 6:**
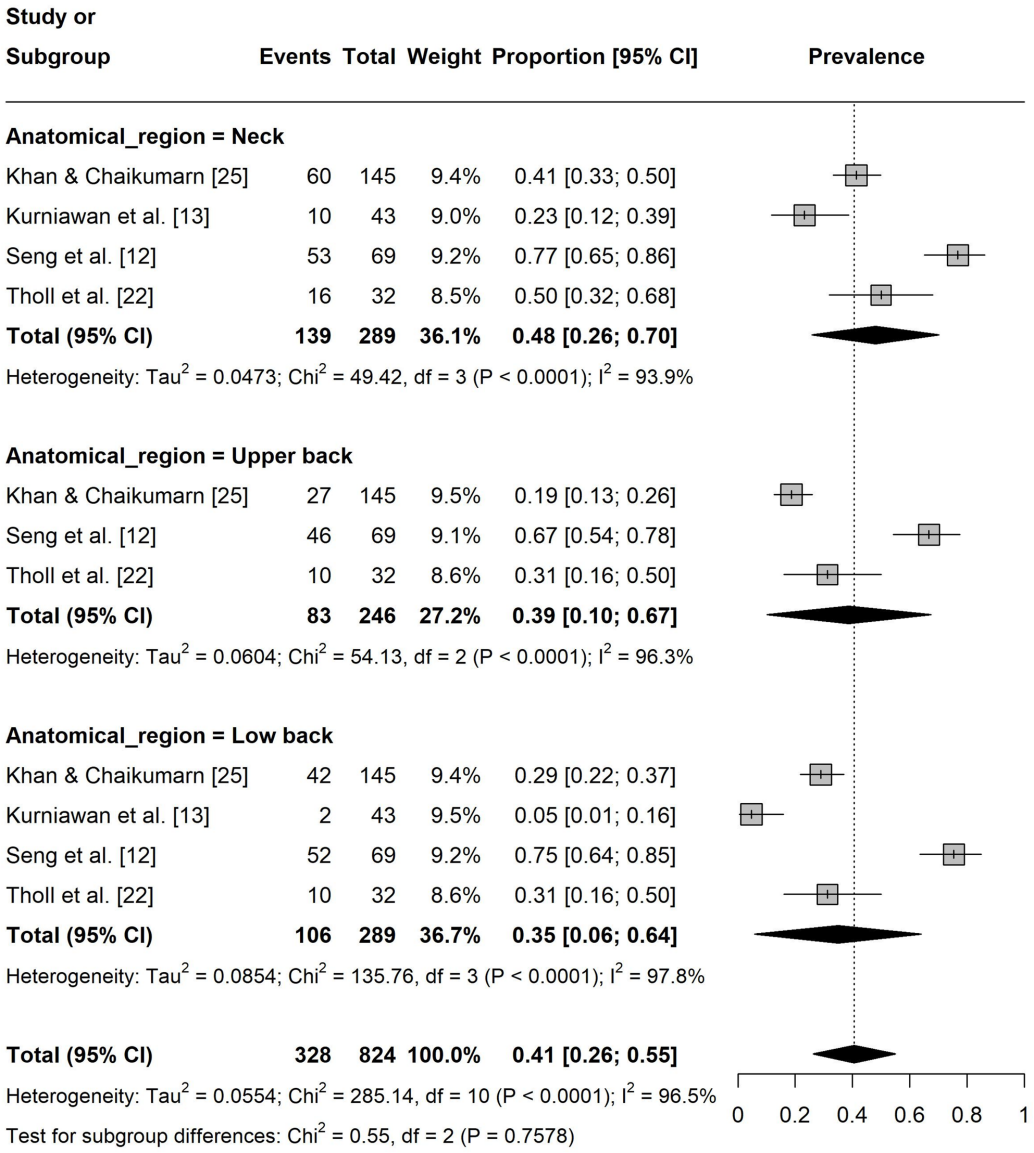
Forest plot of the one-year pain prevalence in the spine. Events = number of participants experiencing pain

Regarding the one-year pain prevalence at specific body sites, the prevalence in lower extremities was 12% ([95% CI: 0.09, 0.15], I² = 0%, k = 8; Fig. 4). The pain prevalence in the upper extremities was 31% ([95% CI: 0.18, 0.44], I² = 96%, k = 12) with shoulder (37%, [95% CI: 0.19, 0.54], I² = 89%, k = 4) and hand/wrist/finger (37%, [95% CI: 0.09, 0.66], I² = 97%, k = 4) being the most affected regions (Fig. 5). For the spine, the prevalence was 41% ([95% CI: 0.26, 0.55], I² = 96%, k = 11) with the neck (48%, [95% CI: 0.26, 0.70], I² = 94%, k = 4) showing the highest prevalence (Fig. 6).

**Fig. 7:**
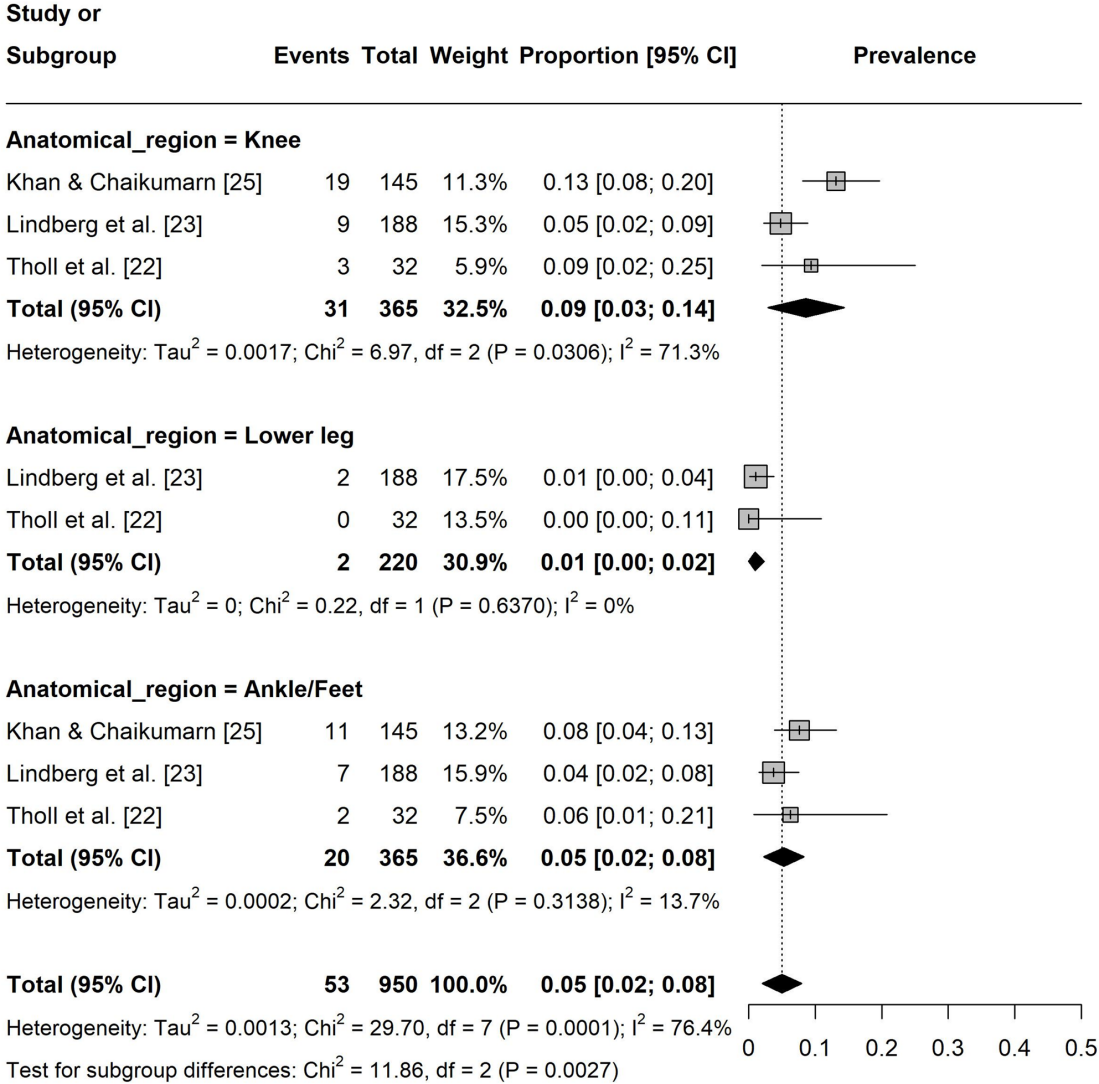
Forest plot of the seven-day pain prevalence in the lower extremities. Events = number of participants experiencing pain

**Fig. 8:**
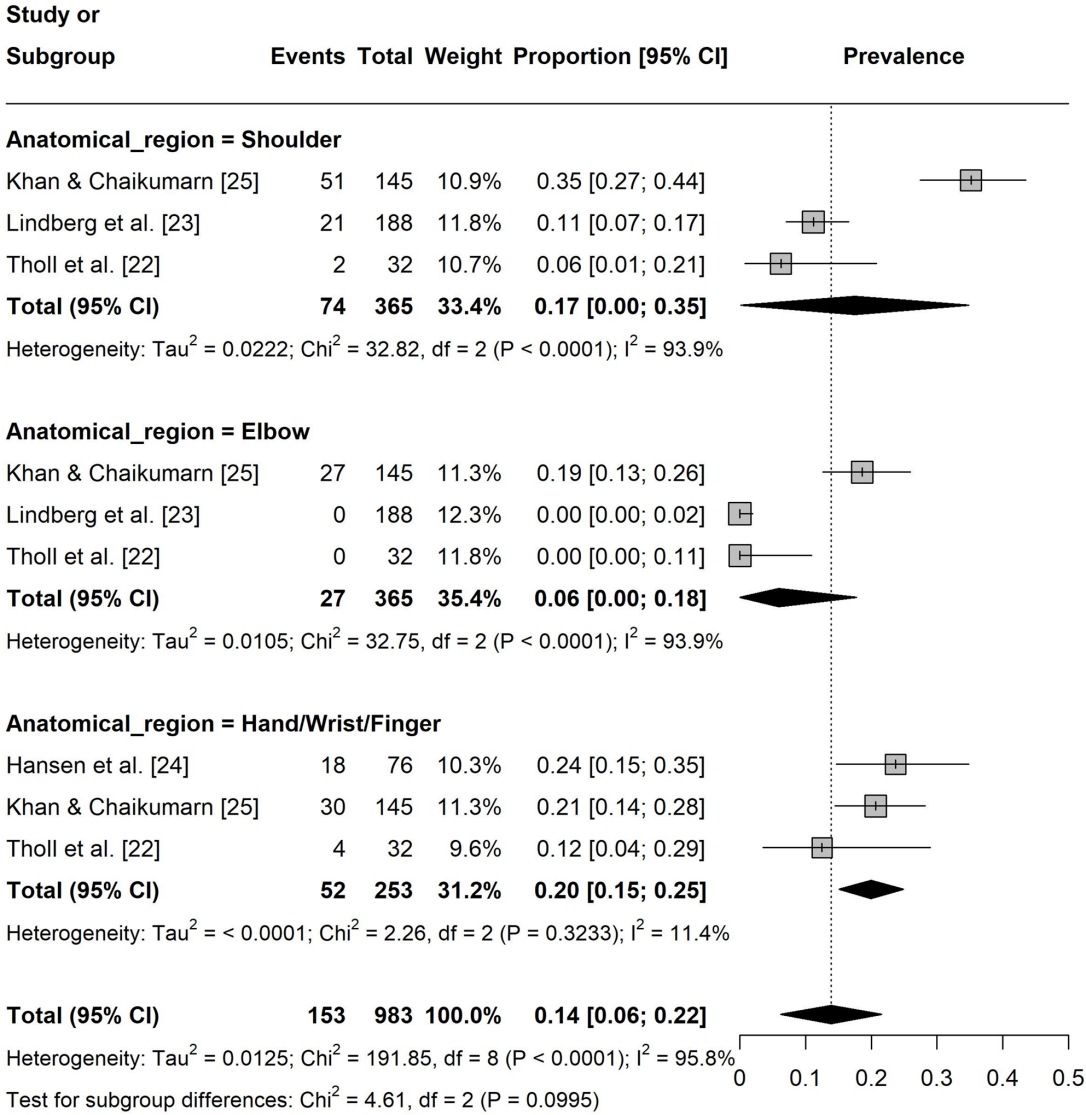
Forest plot of the seven-day pain prevalence in the upper extremities. Events = number of participants experiencing pain

**Fig. 9:**
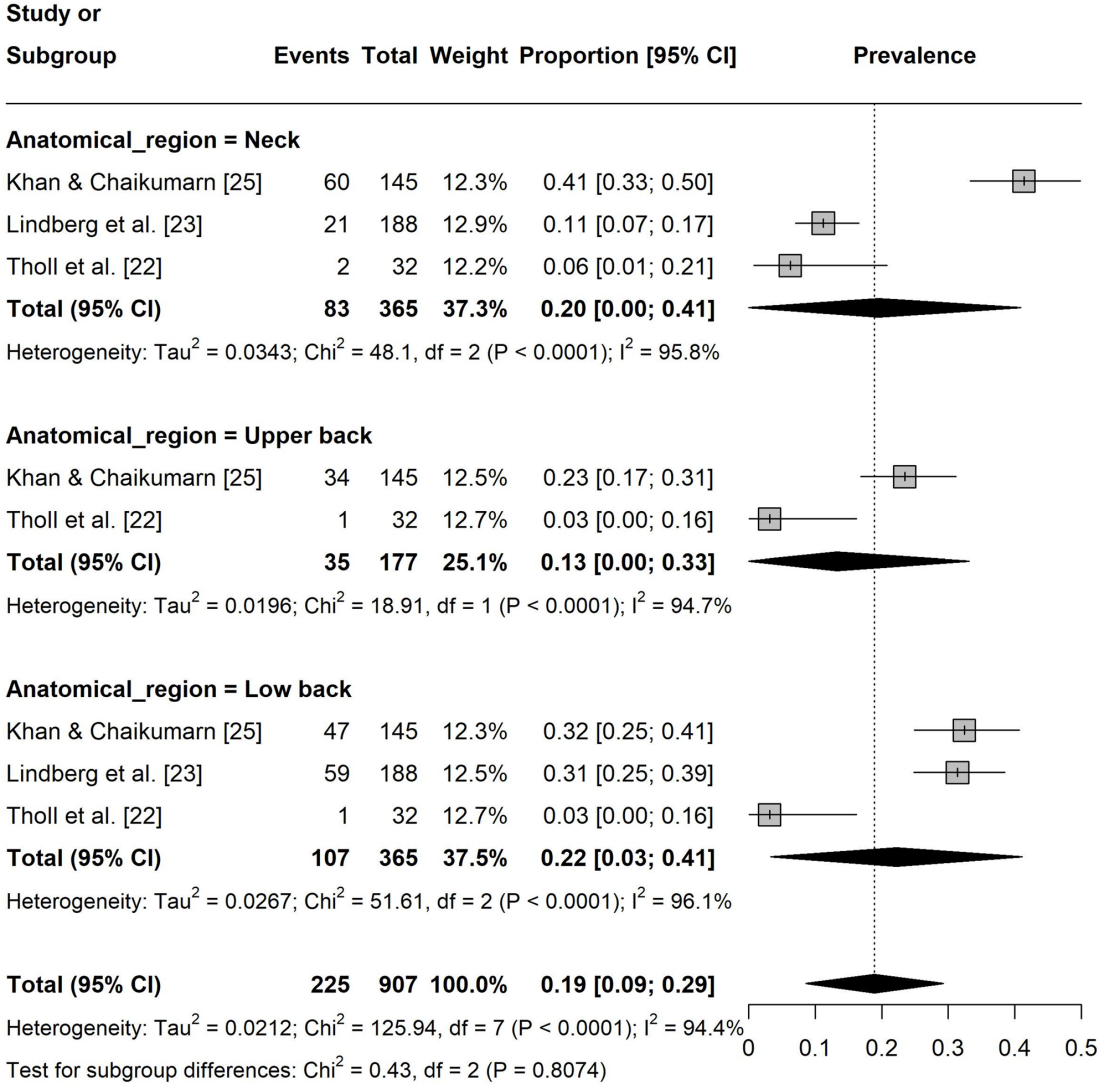
Forest plot of the seven-day pain prevalence in the spine. Events = number of participants experiencing pain

Regarding seven-day pain prevalence, the prevalence in the lower extremities was 5% ([95% CI: 0.02, 0.08], I² = 76%, k = 8 Fig. 7). The prevalence in upper extremities was 14% ([95% CI: 0.06, 0.22], I² = 96%, k = 9) with hand/wrist/finger (20%, [95% CI: 0.15, 0.25], I² = 11%, k = 3) being the most affected region (Fig. 8). For the spine, the prevalence was 19% ([95% CI: 0.09, 0.29], I² = 94%, k = 8) with the lower back (22%, [95% CI: 0.03, 0.41], I² = 96%, k = 3) being the most affected area (Fig. 9).

### Results of Meta-regression

For the exploratory meta-regression analyses, four studies were included for both the one-year prevalence [12, 13, 22, 25] and seven-day prevalence [22–25]. The meta-regression for the seven-day prevalence revealed that both *continent* and *gaming modality* significantly explained the heterogeneity among studies. Specifically, seven-day prevalence was significantly higher in Asian esports players (mean difference: -15.5 [95% CI: -23.4, -7.6], *p* = 0.002) and those using mobile phones for gaming (mean difference: 15.5 [95% CI: 7.6, 23.4], *p* = 0.002). In terms of anatomical region, the spine showed a significantly higher prevalence compared to the lower extremity (mean difference: 13.4 [95% CI: 2.8, 23.9], *p* = 0.012), while differences involving the upper extremity were not statistically significant (Table 2).

**Table 2:** Results for seven-day pain prevalence of subgroup analyses and separate meta-regression analyses based on continent, gaming modality, and anatomical region

	Subgroup analyses			Meta-regression	
	Number of estimates	Pooled estimates (95% CI)	I², %	Mean difference (95% CI)	*p*-value
*Continent*					***0.002***
Asia	8	23.9 [16.0, 31.9]	91.8	–	
Europe	17	8.2 [4.3, 12.1]	95.3	–15.5 [–23.4, –7.6]	
*Gaming modality*					***0.002***
Mobile phone	8	23.9 [16.0, 31.9]	91.8	15.5 [7.6, 23.4]	
Computer-based	17	8.2 [4.3, 12.1]	95.3	–	
*Anatomical region*					*0.193*
Lower extremity	8	5.5 [2.6, 8.4]	77.5	–	
Spine^a^	8	19.6 [9.7, 29.6]	94.0	13.4 [2.8, 23.9]	**0.012**
Upper extremity^a^	9	14.5 [7.0, 22.0]	95.5	8.2 [–2.0, 18.5]	0.116
*Risk of Bias profile*					*0.408*
High risk of bias	8	10.6 [3.1, 18.1]	98.7	–	–
Low risk of bias	17	14.8 [9.0, 20.6]	92.3	4.1 [–5.7, 13.9]	

Bold values show a significant difference (*p* < 0.05). Italicized values show p-value across all subgroups (e.g. continent, gaming modality and anatomical region). For anatomical region lower extremity was used as reference. ^a^comparison between spine and upper extremity revealed no significant difference (*p* = 0.342)

Analyses of the one-year prevalence demonstrated no significant effects for *continent* (*p* = 0.772) and *gaming modality* (*p* = 0.302). However, the parameter anatomical region significantly explained heterogeneity among studies, with the spine (mean difference: 27.2 [95% CI: 9.1, 45.2], *p* = 0.003) and upper extremities (mean difference: 17.9 [95% CI: 1.5, 35.6], *p* = 0.037) showing significantly higher prevalence rates compared to those estimated for the lower extremities (Table 3). As an additional sensitivity analysis, studies were stratified by risk of bias. Studies with only one high or moderate risk of bias were included as low, all others as high. Whereas no significant differences were observed for the seven-day prevalence, one-year prevalence was significantly lower in studies with a low risk of bias profile (mean difference: -15.6 [95% CI: -30.4, -0.8], *p* = 0.039).

**Table 3:** Results for one-year pain prevalence of subgroup analyses and separate meta-regression analyses based on continent, gaming modality, and anatomical region

	Subgroup analyses			Meta-regression	
	Number of estimates	Pooled estimates (95% CI)	I², %	Mean difference (95% CI)	*p*-value
*Continent*					*0.772*
Asia	22	32.3 [22.1, 42.6]	97.4	-	
Europe	9	23.5 [16.0, 31.1]	61.4	-7.9 [-25.2, 9.5]	
*Gaming modality*					*0.302*
Computer-based	9	23.5 [16.0, 31.1]	61.4	-	
Mobile phone	13	19.7 [13.7, 25.8]	89.3	-4.0 [-14.0, 6.0]	
*Anatomical region*					*0.011*
Lower extremity	8	12.5 [9.8,15.1]	0.0	-	
Spine^a^	11	40.8 [26.8, 54.9]	95.7	27.2 [9.1, 45.2]	**0.003**
Upper extremity^a^	12	31.5 [19.0, 44.1]	95.5	17.9 [1.5, 35.6]	**0.037**
*Risk of Bias profile*					0.039
High risk of bias	14	38.7 [23.9, 53.5]	97.0	-	-
Low risk of bias	17	22.3 [17.1, 27.6]	83.9	-15.6 [-30.4, -0.8]	

Bold values show a significant difference (*p* < 0.05). Italicized values show p-value across all subgroups (e.g. continent, gaming modality and anatomical region). For anatomical region, lower extremity was used as reference. ^a^comparison between spine and upper extremity revealed no significant difference (*p* = 0.485)

### Results of Studies not Included in Meta-analysis

Seven studies [26–32] could not be included in the meta-analysis due to lack of specified time prevalence or sufficient studies on the respective time prevalence (e.g. 6 months, 3 months). In these studies, only one study reported an overall pain prevalence of 62.7% within three months [30]. In four studies, the back was the most frequently reported mentioned painful area [26, 28, 31, 32]. In two studies [29, 30], headaches were reported as the most frequently cited area of complaint, while neck pain was identified as the most common issue in one study [27]. These areas of the body are repeatedly listed among the three most affected areas in all studies. In addition, the wrist was among the three most affected body parts in three studies [28, 30, 32], the fingers in one [27], and the shoulders in another one [31]. When a distinction was made between the lower and the upper back, the lower back had a higher prevalence in all but one study. This was the case in three studies [26, 29, 30].

### Risk of Bias and Quality Assessment of Studies

An overview of the risk of bias assessment of all included studies and their individual scores is presented in Supplementary Table 4 in the supplementary information. In the first domain with regard to the target population, all except one study [28] had a high risk of bias, as most of the studies only acquired a certain subset of esports players (e.g., only mobile players, professionals, men, specific age group etc.) rather than the broader population of esports players. In the second domain, eleven out of thirteen studies had a high risk of bias. Ideally, according to the risk of bias tool [17], a list of sampling units of the target population should be created in advance, from which the study sample is subsequently drawn. With the exception of two studies [25, 30], this was not conducted in any of them. The results for domain three, regarding random selection, were similar. Only the two studies that provided a list of potential participants for inclusion employed a random recruitment strategy and, therefore, were assigned a low risk of bias [25, 30]. Eight studies had a low response-rate (< 75%) or did not report it at all, resulting in a high risk of non-response bias [12, 13, 24, 26, 28, 29, 31, 32]. In domain five, all studies had a low risk, as the data were all collected directly from the participants. In the case definition category, five studies were rated with a high risk, because they did not provide a clear definition of the outcome, used multiple terms for it (e.g., musculoskeletal symptoms and pain) or did not report a specific time prevalence [12, 13, 22, 26, 32]. In eight of thirteen studies, no validated study instrument was used for the main outcome pain, for which they were rated with a high risk of bias [23, 24, 26–29, 31, 32]. In category eight, all were rated as having a low risk, as all participants were asked the same questions about the outcome in the individual studies. With regard to time prevalence, six out of thirteen studies had a high risk of bias, as they either used long time periods (≥ 3 months) or no time prevalence was stated at all [12, 13, 26, 27, 30, 32]. In category ten, only one study was rated with a high risk of bias, as it excluded women from the statistical analyses and therefore only prevalence rates for men were provided [29].

## Discussion

This is the first meta-analysis to examine the prevalence of pain overall and in different body regions among esports players and, thus, provides an important insight into the extent to which esports players are at risk of being affected by pain.

Regarding the first research question, the overall one-year prevalence of pain among esports players is 73%, whereas the seven-day prevalence stands at 44%. It is important to note that these prevalence values are based on only two studies (one-year prevalence) and three studies (seven-day prevalence), respectively. According to this, almost three quarters have experienced pain within a year and almost half within a week. Given that esports players represent a young target group, this can be considered a high prevalence of pain. In the general population, an age- and sex-adjusted pain prevalence of 27.5% within the last 30 days was reported [33]. However, it should be noted that this study exclusively examined moderate to severe pain, with mild pain not being included. Furthermore, the referenced [33] study focused on individuals over the age of 25, which complicates comparisons, as older individuals are generally more likely to experience pain than younger populations [34]. Otherwise, studies tended to investigate chronic pain in the general population, which also makes a direct comparison difficult. This is defined as pain that has persisted for at least 3 months. Studies reported a prevalence of chronic pain of 11–14% among adolescents and young adults [35, 36]. Although these values are lower than those reported in this meta-analysis, it is important to note that every instance of pain was considered in this meta-analysis. Since chronic pain is more clinically relevant and would provide a better basis for comparison with the general population, it is recommended that future research also examines chronic pain states in esports players. The high prevalence of pain among esports players observed in this study might be partly related to the sedentary and inactive nature of video gaming. Previous research in the general population has highlighted associations between sedentary behavior and musculoskeletal pain across different age groups [37]. However, it should be noted that much of this evidence is based on cross-sectional studies, limiting causal interpretation. For instance, Montgomery et al. (2024) [38] reported that sedentary time was associated with spinal pain in adolescents, but this association did not persist when only longitudinal data were considered. In the context of esports players, longer playing times might be associated with a higher risk of experiencing pain, particularly when playing for more than three hours [8]. Esports players typically have extended playing times, as reflected in the included studies, where an average of three hours per day was the minimum reported playing time. This prolonged engagement in video gaming could be accompanied by an increased frequency of pain, with two studies reporting that almost half of the participants stated that they experienced pain daily or several times a week [23, 24].

Pain manifestation can occur in different parts of the body, which is the focus of the second research question. For both the seven-day and the one-year prevalence, the spine is most frequently affected, followed by the upper extremities. In the lower extremities, pain is reported less frequently. Furthermore, the findings indicate that the lower back is more frequently affected by pain than the neck when considering the seven-day prevalence. In contrast, the one-year prevalence is highest in the neck, followed by the upper back. It is important to note, however, that the number of studies differs in both prevalence periods for the upper back. Nevertheless, these findings align with existing literature, which identified back and neck pain as the most commonly reported areas of complaints [8, 39]. Prolonged sitting in monotonous postures has been suggested as a potential contributing factor to musculoskeletal pain among esports players [11, 40]. A common posture during gaming involves a flexed neck with the head slightly placed forward, which places additional strain on the cervical extensor muscles, leading to increased fatigue over time [40, 41]. According to Emara et al. (2020), this can cause axial back pain, which can range from the neck through the lumbar regions [11]. In addition to maintaining the same posture for long periods of time, other factors can also contribute to pain in esports players, such as suboptimal ergonomics, repetitive upper extremity movements, long training sessions with infrequent breaks, or lower overall physical fitness [39]. Pain is unlikely to result from a single determinant but rather from the interaction of multiple contributing factors. Alongside physical risk factors, psychosocial influences may also play a significant role in the development of pain. One example of this could be poor sleep quality [42], which is often noticeable among esports players [43]. Compared to the German population over the age of 18, the one-year prevalence rates are relatively similar. However, esports players exhibit a higher prevalence of neck and upper back pain while experiencing lower rates of lower back pain [44]. The prevalence of pain can vary depending on the country and age [33]. This is also supported by the results of the meta-regression, in which esports players on the Asian continent show significantly higher pain prevalence than esports players in Europe. However, this represents a significant generalization and should not be oversimplified, as the studies included in the meta-analysis and meta-regression focus only on specific subregions within each respective continent. Within the general Asian population, substantial differences in point prevalence exist between individual countries (e.g., Philippines, about 35% vs. China, about 10%) [33]. Similarly, in Europe, prevalence rates vary among countries, albeit generally lower (e.g., Finland, about 22% vs. Sweden, about 28%) [33]. However, when countries are grouped by continents, the overall pain prevalence appears slightly elevated in Europe (34%) compared to Southeast Asia (30%) [33]. Furthermore, pain prevalence rates may differ substantially within a single country, depending on factors such as area of residence, particular in geographically large countries like China. A study by Rafferty et al. (2021) observed a significantly higher prevalence of chronic pain in rural areas (31%) compared to urban regions (20%) in North Carolina, USA [45]. It also needs to be stated that only a limited number of studies could be included for the comparisons of pain prevalence between continents, which restricts the ability to draw conclusive inferences.

Regarding pain in the upper extremity, the wrist/hand/finger is most frequently affected area in both the seven-day prevalence and the one-year prevalence, followed by the shoulder. In addition to back and neck pain, wrist pain is also often frequently mentioned in the literature [7, 11, 40]. Moreover, upper extremity pain is reported to be responsible for esports players having to end their careers at a relatively young age due to pain [7]. This is often caused by repetitive strain and overuse of the structures of the upper extremities, as playing video games requires fast, repetitive movements of the fingers, hands, and wrists [7, 39]. This constant strain without sufficient regeneration often leads to injuries such as tendinopathies or carpal tunnel syndrome [39]. Compared to the general population (19.1%), the prevalence of wrist pain within a year is noticeably higher in esports players [46]. While general physical activity guidelines are equally applicable to esports players, the unique demands of competitive gaming may call for complementary, targeted strategies. Tailored exercise interventions, such as strengthening or mobility programs, may help for prevention of upper extremity musculoskeletal disorders [11, 47]. Nevertheless, further research is needed, particularly to investigate the load on the upper extremities in esports players in order to develop appropriate strategies for load management. In addition, other components may also play an important role in pain prevention and management, including ergonomic adjustments, structured breaks, sleep hygiene, and educational approaches [11]. A multifaceted strategy that integrates these aspects may therefore be most effective in reducing pain prevalence and supporting the health of esports players. Furthermore, future research could explore the extent to which esports players use analgesics. Among the included studies, only one investigated this aspect, reporting a relatively low use of pain medication (9.6%) [23].

Although it was not possible to carry out an analysis of pain intensity due to a lack of data, the studies that recorded this parameter showed a low pain intensity of 3–4 out of 10 on a numerical rating scale [23, 24]. Despite pain scores of 3 to 4 being classified as mild pain according to the International Association for the Study of Pain [48], it is concerning that in two studies, more than half of the participants reported experiencing pain daily or for several times a week, with a small proportion even reporting activity limitations as a result [23, 24]. To better assess the severity and impact of pain, future studies should include these two parameters in their surveys. Additionally, it would be beneficial to investigate the persistence of pain among esports player in order to assess chronic pain conditions.

As already called for in the literature, further prevalence data within methodologically rigorous designs are needed through future research [49]. This should prioritize the use of standardized and reliable questionnaires to assess pain to ensure greater homogeneity among studies. This includes the adoption of consistent terminology. Many studies have employed various synonyms for pain, such as musculoskeletal discomfort or physical symptoms, which complicates the interpretation of findings. For instance, musculoskeletal discomfort may encompass more than just pain, making it unclear what specifically was assessed. Moreover, it is crucial to examine and precisely define prevalence periods during which pain was experienced to establish an accurate time prevalence. As demonstrated by the results, there is, unsurprisingly, an enormous difference between asking about current pain, pain within the last seven days, or pain within the past year. Without consideration of this temporal context, the results cannot be accurately interpreted. Future research should also focus on examining pain prevalence separately by gaming device, as the results of the meta-regression demonstrate that mobile players have significantly higher prevalence rates than computer gamers. Other gaming modalities, such as exergames, were excluded from this review and further research is needed to investigate the prevalence of pain in these specific forms of gaming. It is also possible that players may experience pain in different anatomical regions depending on the gaming device they use, highlighting the need for targeted interventions tailored to the specific demands of each gaming device.

It is important to note that despite the subgroup analyses, heterogeneity remained high in most models. This suggests that additional, unmeasured moderators may contribute to the variability between studies. Potential factors might include differences in time spent playing video games, lifetime gaming experience, regular physical activity patterns, age and sex distribution, as well as variation in the instruments used to assess pain. Due to limited and inconsistent reporting across the included studies, these moderators could not be examined in the present analysis. Given the high degree of heterogeneity in the results, the findings should be interpreted with caution.

### Limitations of the Included Studies

The results should be interpreted with caution due to some concerns regarding the risk of bias. Due to a lack of definition or no indication of time prevalence, some studies were accorded a high risk of bias. In addition, the participants in the studies were often not a true representation of the actual target group of esports players, as the target group was often narrowed down further (e.g., only mobile players or only participants in a tournament). The majority of the included studies also had a high risk of bias due to the fact that participants were not randomly selected from a predefined list. In most cases, convenience sampling was used, by which participants were selected based on their response to advertisements via social media or similar platforms. Most studies did not provide a precise definition of esports players, making it difficult to distinguish between recreational and professional participants. Moreover, sample sizes varied substantially across studies, ranging from as few as 32 to as many as 188 participants, which further limits the comparability and generalizability of findings. Another important limitation is that the severity of pain was rarely assessed in the included studies. Most investigations reported only the presence or absence of pain without differentiating between mild, moderate, or severe pain. Consequently, the clinical relevance of the reported prevalence rates remains uncertain. Lastly, there was a lack of differentiation in the included studies between different types of pain, such as acute or chronic. All studies only referred only to “pain” without specifying its origin or duration. This lack of specificity limits the interpretability of the findings, as chronic pain is generally considered more clinically relevant due to its prolonged impact on quality of life and functionality [50, 51]. Future research should distinguish between these pain types to provide a more nuanced understanding of pain prevalence in esports players. Nevertheless, there were also studies that had a low risk of bias across almost all categories. However, the proportion of lower quality studies predominated, highlighting the need for more well-designed research to be conducted.

### Limitations of the Current Review

This meta-analysis is subject to several limitations. The strict study inclusion criterion of only including studies that explicitly mentioned pain resulted in a limited number of studies being included in this meta-analysis. However, studies should explicitly define the specific outcomes being assessed. Since pain differs distinctly from discomfort or injury, conflating these terms under a single category could introduce significant bias. The limited number of studies also resulted in a relatively small total sample size, particularly concerning the overall prevalence. Additionally, the high degree of heterogeneity across studies in terms of design and assessment methods further limited the interpretability of the results and prevented meaningful subgroup analyses (e.g. age groups, sex or level of play). Finally, the lack of standardized questionnaires with clearly defined outcomes and time periods reduces comparability across studies. Together, these limitations highlight the urgent need for more rigorous and standardized research to enable a more representative and nuanced analysis of pain prevalence in esports players.

## Conclusion

This meta-analysis provides the first comprehensive overview of pain prevalence in esports players, suggesting high one-year (73%) and seven-day (44%) prevalence rates, albeit including only a limited number of studies of high heterogeneity. The findings highlight the spine and upper extremities, particularly the neck, low back and wrist, as the most commonly affected regions. Yet, the heterogeneity and low number of the included studies limits the interpretability of the results. In addition, it is recommended to differentiate between acute and chronic pain in the future, as the latter possesses a higher clinical relevance. Standardized assessments with clear definitions of pain and time prevalences are essential for future research to ensure comparability and enhance understanding of pain in this population. Targeted prevention strategies, such as load management, ergonomic interventions, and exercise programs are urgently needed to mitigate these risks for pain.

## Supplementary Information

Below is the link to the electronic supplementary material.

Supplementary Material 1.

Supplementary Material 2.

## Data Availability

The datasets used and analyzed during the current study are available from the corresponding author on reasonable request.
