# Supplementary material for: Do Esports Players Experience Pain? Pain Prevalence of Esports Players: a Systematic Review and Meta-analysis
Source: Sports Med Open. 2026 Jan 3;12:1. doi: 10.1186/s40798-025-00971-1 (PMC12764727; doi:10.1186/s40798-025-00971-1)
Supplement: Supplementary file 2 — Supplementary Material 2. [file 40798_2025_971_MOESM2_ESM.docx]

**Table S1** Search strategy and search strings used in PUBMED

| PUBMED | “pain” | “esports” | Hits |
| --- | --- | --- | --- |
| Domain strings | “pain”[Title/Abstract] OR “physical health”[Title/Abstract] OR “physical symptoms”[Title/Abstract] OR “musculoskeletal”[Title/Abstract] OR “physical problems” [Title/Abstract] OR “health issues” [Title/Abstract] | “esports” [Title/Abstract] OR “esport” [Title/Abstract] OR “gaming” [Title/Abstract] OR “videogames” [Title/Abstract] OR “videogame” [Title/Abstract] OR “e-athlete” [Title/Abstract] OR “video game player” [Title/Abstract] OR “e-gamer” [Title/Abstract] OR “gamer”[Title/Abstract] |  |
| Final search string combinations | (pain[Title/Abstract]) AND ("esports"[Title/Abstract])  (pain[Title/Abstract]) AND ("esport"[Title/Abstract])  (Pain[Title/Abstract]) AND ("gaming"[Title/Abstract])  (Pain[Title/Abstract]) AND ("videogames"[Title/Abstract])  (Pain[Title/Abstract]) AND ("videogame"[Title/Abstract])  (Pain[Title/Abstract]) AND ("digital games"[Title/Abstract])  (Pain[Title/Abstract]) AND ("e-athlete"[Title/Abstract])  (Pain[Title/Abstract]) AND ("video player"[Title/Abstract])  (Pain[Title/Abstract]) AND ("e-gamer"[Title/Abstract])  (Pain[Title/Abstract]) AND ("gamer"[Title/Abstract])  (pain[Title/Abstract]) AND ("video game player"[Title/Abstract])  ("Physical health"[Title/Abstract]) AND ("esports"[Title/Abstract])  ("Physical health"[Title/Abstract]) AND ("esport"[Title/Abstract])  ("Physical health"[Title/Abstract]) AND ("gaming"[Title/Abstract])  ("Physical health"[Title/Abstract]) AND ("videogames"[Title/Abstract])  ("Physical health"[Title/Abstract]) AND ("videogame"[Title/Abstract])  ("Physical health"[Title/Abstract]) AND ("e-athlete"[Title/Abstract])  ("Physical health"[Title/Abstract]) AND ("video game player"[Title/Abstract])  ("Physical health"[Title/Abstract]) AND ("e-gamer"[Title/Abstract])  ("Physical health"[Title/Abstract]) AND ("gamer"[Title/Abstract])  ("Physical symptoms"[Title/Abstract]) AND ("esports"[Title/Abstract])  ("Physical symptoms"[Title/Abstract]) AND ("esport"[Title/Abstract])  ("Physical symptoms"[Title/Abstract]) AND ("gaming"[Title/Abstract])  ("Physical symptoms"[Title/Abstract]) AND ("videogames"[Title/Abstract])  ("Physical symptoms"[Title/Abstract]) AND ("videogame"[Title/Abstract])  ("Physical symptoms"[Title/Abstract]) AND ("e-athlete"[Title/Abstract])  ("Physical symptoms"[Title/Abstract]) AND ("video game player"[Title/Abstract])  ("Physical symptoms"[Title/Abstract]) AND ("e-gamer"[Title/Abstract])  ("Physical symptoms"[Title/Abstract]) AND ("gamer"[Title/Abstract])  ("musculoskeletal"[Title/Abstract]) AND ("esports"[Title/Abstract])  ("musculoskeletal"[Title/Abstract]) AND ("esport"[Title/Abstract])  ("musculoskeletal"[Title/Abstract]) AND ("gaming"[Title/Abstract])  ("musculoskeletal"[Title/Abstract]) AND ("videogames"[Title/Abstract])  ("musculoskeletal"[Title/Abstract]) AND ("videogame"[Title/Abstract])  ("musculoskeletal"[Title/Abstract]) AND ("e-athlete"[Title/Abstract])  ("musculoskeletal"[Title/Abstract]) AND ("video game player"[Title/Abstract])  ("musculoskeletal"[Title/Abstract]) AND ("e-gamer"[Title/Abstract])  ("musculoskeletal"[Title/Abstract]) AND ("gamer"[Title/Abstract])  ("physical problems"[Title/Abstract]) AND ("esports"[Title/Abstract])  ("physical problems"[Title/Abstract]) AND ("esport"[Title/Abstract])  ("physical problems"[Title/Abstract]) AND ("gaming"[Title/Abstract])  ("physical problems"[Title/Abstract]) AND ("videogames"[Title/Abstract])  ("physical problems"[Title/Abstract]) AND ("videogame"[Title/Abstract])  ("physical problems"[Title/Abstract]) AND ("e-athlete"[Title/Abstract])  ("physical problems"[Title/Abstract]) AND ("video game player"[Title/Abstract])  ("physical problems"[Title/Abstract]) AND ("e-gamer"[Title/Abstract])  ("physical problems"[Title/Abstract]) AND ("gamer"[Title/Abstract])  ("health issues"[Title/Abstract]) AND ("esports"[Title/Abstract])  ("health issues"[Title/Abstract]) AND ("esport"[Title/Abstract])  ("health issues"[Title/Abstract]) AND ("gaming"[Title/Abstract])  ("health issues"[Title/Abstract]) AND ("videogames"[Title/Abstract])  ("health issues"[Title/Abstract]) AND ("videogame"[Title/Abstract])  ("health issues"[Title/Abstract]) AND ("e-athlete"[Title/Abstract])  ("health issues"[Title/Abstract]) AND ("video game player"[Title/Abstract])  ("health issues"[Title/Abstract]) AND ("e-gamer"[Title/Abstract])  ("health issues"[Title/Abstract]) AND ("gamer"[Title/Abstract]) | | 9  2  138  15  24  3  0  0  0  3  1  9  2  56  8  5  0  1  0  2  1  0  8  0  0  0  0  0  0  14  1  52  8  3  0  1  0  2  0  0  2  0  0  0  0  0  0  6  3  42  7  2  0  0  0  1 |
| Hits in total | 431 | |  |

**Table S2** Search strategy and search strings used in Google Scholar

| Google Scholar | “pain” | “esports” | Hits |
| --- | --- | --- | --- |
| Domain strings | “pain”[Title/Abstract] OR “physical health”[Title/Abstract] OR “physical symptoms”[Title/Abstract] OR “musculoskeletal”[Title/Abstract] OR “physical problems” [Title/Abstract] OR “health issues” [Title/Abstract] | “esports” [Title/Abstract] OR “esport” [Title/Abstract] OR “gaming” [Title/Abstract] OR “videogames” [Title/Abstract] OR “videogame” [Title/Abstract] OR “e-athlete” [Title/Abstract] OR “video game player” [Title/Abstract] OR “e-gamer” [Title/Abstract] OR “gamer”[Title/Abstract] |  |
| Final search string combinations | (pain[Title/Abstract]) AND ("esports"[Title/Abstract])  (pain[Title/Abstract]) AND ("esport"[Title/Abstract])  (Pain[Title/Abstract]) AND ("gaming"[Title/Abstract])  (Pain[Title/Abstract]) AND ("videogames"[Title/Abstract])  (Pain[Title/Abstract]) AND ("videogame"[Title/Abstract])  (Pain[Title/Abstract]) AND ("digital games"[Title/Abstract])  (Pain[Title/Abstract]) AND ("e-athlete"[Title/Abstract])  (Pain[Title/Abstract]) AND ("video player"[Title/Abstract])  (Pain[Title/Abstract]) AND ("e-gamer"[Title/Abstract])  (Pain[Title/Abstract]) AND ("gamer"[Title/Abstract])  (pain[Title/Abstract]) AND ("video game player"[Title/Abstract])  ("Physical health"[Title/Abstract]) AND ("esports"[Title/Abstract])  ("Physical health"[Title/Abstract]) AND ("esport"[Title/Abstract])  ("Physical health"[Title/Abstract]) AND ("gaming"[Title/Abstract])  ("Physical health"[Title/Abstract]) AND ("videogames"[Title/Abstract])  ("Physical health"[Title/Abstract]) AND ("videogame"[Title/Abstract])  ("Physical health"[Title/Abstract]) AND ("e-athlete"[Title/Abstract])  ("Physical health"[Title/Abstract]) AND ("video game player"[Title/Abstract])  ("Physical health"[Title/Abstract]) AND ("e-gamer"[Title/Abstract])  ("Physical health"[Title/Abstract]) AND ("gamer"[Title/Abstract])  ("Physical symptoms"[Title/Abstract]) AND ("esports"[Title/Abstract])  ("Physical symptoms"[Title/Abstract]) AND ("esport"[Title/Abstract])  ("Physical symptoms"[Title/Abstract]) AND ("gaming"[Title/Abstract])  ("Physical symptoms"[Title/Abstract]) AND ("videogames"[Title/Abstract])  ("Physical symptoms"[Title/Abstract]) AND ("videogame"[Title/Abstract])  ("Physical symptoms"[Title/Abstract]) AND ("e-athlete"[Title/Abstract])  ("Physical symptoms"[Title/Abstract]) AND ("video game player"[Title/Abstract])  ("Physical symptoms"[Title/Abstract]) AND ("e-gamer"[Title/Abstract])  ("Physical symptoms"[Title/Abstract]) AND ("gamer"[Title/Abstract])  ("musculoskeletal"[Title/Abstract]) AND ("esports"[Title/Abstract])  ("musculoskeletal"[Title/Abstract]) AND ("esport"[Title/Abstract])  ("musculoskeletal"[Title/Abstract]) AND ("gaming"[Title/Abstract])  ("musculoskeletal"[Title/Abstract]) AND ("videogames"[Title/Abstract])  ("musculoskeletal"[Title/Abstract]) AND ("videogame"[Title/Abstract])  ("musculoskeletal"[Title/Abstract]) AND ("e-athlete"[Title/Abstract])  ("musculoskeletal"[Title/Abstract]) AND ("video game player"[Title/Abstract])  ("musculoskeletal"[Title/Abstract]) AND ("e-gamer"[Title/Abstract])  ("musculoskeletal"[Title/Abstract]) AND ("gamer"[Title/Abstract])  ("physical problems"[Title/Abstract]) AND ("esports"[Title/Abstract])  ("physical problems"[Title/Abstract]) AND ("esport"[Title/Abstract])  ("physical problems"[Title/Abstract]) AND ("gaming"[Title/Abstract])  ("physical problems"[Title/Abstract]) AND ("videogames"[Title/Abstract])  ("physical problems"[Title/Abstract]) AND ("videogame"[Title/Abstract])  ("physical problems"[Title/Abstract]) AND ("e-athlete"[Title/Abstract])  ("physical problems"[Title/Abstract]) AND ("video game player"[Title/Abstract])  ("physical problems"[Title/Abstract]) AND ("e-gamer"[Title/Abstract])  ("physical problems"[Title/Abstract]) AND ("gamer"[Title/Abstract])  ("health issues"[Title/Abstract]) AND ("esports"[Title/Abstract])  ("health issues"[Title/Abstract]) AND ("esport"[Title/Abstract])  ("health issues"[Title/Abstract]) AND ("gaming"[Title/Abstract])  ("health issues"[Title/Abstract]) AND ("videogames"[Title/Abstract])  ("health issues"[Title/Abstract]) AND ("videogame"[Title/Abstract])  ("health issues"[Title/Abstract]) AND ("e-athlete"[Title/Abstract])  ("health issues"[Title/Abstract]) AND ("video game player"[Title/Abstract])  ("health issues"[Title/Abstract]) AND ("e-gamer"[Title/Abstract])  ("health issues"[Title/Abstract]) AND ("gamer"[Title/Abstract]) | | 35  43  827  156  147  112  3  12  0  82  2  26  13  352  70  52  4  2  0  46  0  3  54  9  10  0  0  0  2  18  28  207  48  42  3  1  0  20  0  1  27  7  3  0  0  0  1  16  2  329  48  39  1  2  0  25 |
| Hits in total | 2930 | |  |

**Table S3** Search strategy and search strings used in Web of Science

| Web of Science | “pain” | “esports” | Hits |
| --- | --- | --- | --- |
| Domain strings | “pain”[Title/Abstract] OR “physical health”[Title/Abstract] OR “physical symptoms”[Title/Abstract] OR “musculoskeletal”[Title/Abstract] OR “physical problems” [Title/Abstract] OR “health issues” [Title/Abstract] | “esports” [Title/Abstract] OR “esport” [Title/Abstract] OR “gaming” [Title/Abstract] OR “videogames” [Title/Abstract] OR “videogame” [Title/Abstract] OR “e-athlete” [Title/Abstract] OR “video game player” [Title/Abstract] OR “e-gamer” [Title/Abstract] OR “gamer”[Title/Abstract] |  |
| Final search string combinations | (AB=(pain)) AND AB=("esport")  (AB=(pain)) AND AB=("esports")  (AB=(pain)) AND AB=("gaming")  (AB=(pain)) AND AB=("videogames")  (AB=(pain)) AND AB=("videogame")  (AB=(pain)) AND AB=("e-athlete")  (AB=(pain)) AND AB=("video game player")  (AB=(pain)) AND AB=("e-gamer")  (AB=(pain)) AND AB=("gamer")  (AB=("physical health")) AND AB=("esport")  (AB=("physical health")) AND AB=("esports")  (AB=("physical health")) AND AB=("gaming")  (AB=("physical health")) AND AB=("videogames")  (AB=("physical health")) AND AB=("videogame")  (AB=("physical health")) AND AB=("e-athlete")  (AB=("physical health")) AND AB=("video game player")  (AB=("physical health")) AND AB=("e-gamer")  (AB=("physical health")) AND AB=("gamer")  (AB=("physical symptoms")) AND AB=("esport")  (AB=("physical symptoms")) AND AB=("esports")  (AB=("physical symptoms")) AND AB=("gaming")  (AB=("physical symptoms")) AND AB=("videogames")  (AB=("physical symptoms")) AND AB=("videogame")  (AB=("physical symptoms")) AND AB=("e-athlete")  (AB=("physical symptoms")) AND AB=("video game player")  (AB=("physical symptoms")) AND AB=("e-gamer")  (AB=("physical symptoms")) AND AB=("gamer")  (AB=("musculoskeletal")) AND AB=("esport")  (AB=("musculoskeletal")) AND AB=("esports")  (AB=("musculoskeletal")) AND AB=("gaming")  (AB=("musculoskeletal")) AND AB=("videogames")  (AB=("musculoskeletal")) AND AB=("videogame")  (AB=("musculoskeletals")) AND AB=("e-athlete")  (AB=("musculoskeletal")) AND AB=("video game player")  (AB=("musculoskeletal")) AND AB=("e-gamer")  (AB=("musculoskeletal")) AND AB=("gamer")  (AB=("physical problems")) AND AB=("esport")  (AB=("physical problems")) AND AB=("esports")  (AB=("physical problems")) AND AB=("gaming")  (AB=("physical problems")) AND AB=("videogames")  (AB=("physical problems")) AND AB=("videogame")  (AB=("physical problems")) AND AB=("e-athlete")  (AB=("physical problems")) AND AB=("video game player")  (AB=("physical problems")) AND AB=("e-gamer")  (AB=("physical problems")) AND AB=("gamer")  (AB=("health issues")) AND AB=("esport")  (AB=("health issues")) AND AB=("esports")  (AB=("health issues")) AND AB=("gaming")  (AB=("health issues")) AND AB=("videogames")  (AB=("health issues")) AND AB=("videogame")  (AB=("health issues")) AND AB=("e-athlete")  (AB=("health issues")) AND AB=("video game player")  (AB=("health issues")) AND AB=("e-gamer")  (AB=("health issues")) AND AB=("gamer") | | 7  14  141  16  15  0  1  0  2  1  11  68  9  5  0  1  0  3  0  1  10  0  1  0  0  0  0  5  13  52  10  1  0  1  0  1  0  0  2  0  0  0  0  0  0  1  5  51  6  4  0  0  0  2 |
| Hits in total | 460 | |  |

**Table S4** Overview of the risk of bias according to Hoy et al. (2012)

| **Author (year)** | **1)** | **2)** | **3)** | **4)** | **5)** | **6)** | **7)** | **8)** | **9)** | **10)** |
| --- | --- | --- | --- | --- | --- | --- | --- | --- | --- | --- |
| Bahrilli (2022) | HIGH | HIGH | HIGH | HIGH | LOW | HIGH | HIGH | LOW | HIGH | LOW |
| DiFrancicsco-Donoghue (2019) | HIGH | HIGH | HIGH | HIGH | LOW | HIGH | HIGH | LOW | HIGH | LOW |
| Ekefjärd (2024) | HIGH | LOW | LOW | LOW | LOW | LOW | LOW | LOW | HIGH | LOW |
| Gaasedal (2023) | HIGH | HIGH | HIGH | HIGH | LOW | LOW | HIGH | LOW | LOW | LOW |
| Hansen (2024) | HIGH | HIGH | HIGH | HIGH | LOW | LOW | HIGH | LOW | LOW | LOW |
| Khan (2024) | HIGH | LOW | LOW | LOW | LOW | LOW | LOW | LOW | LOW | LOW |
| Kurniawan (2024) | HIGH | HIGH | HIGH | HIGH | LOW | HIGH | LOW | LOW | HIGH | LOW |
| Lam (2022) | HIGH | HIGH | HIGH | LOW | LOW | LOW | HIGH | LOW | HIGH | LOW |
| Lindberg (2020) | HIGH | HIGH | HIGH | LOW | LOW | LOW | HIGH | LOW | LOW | LOW |
| Mariano (2024) | LOW | HIGH | HIGH | HIGH | LOW | LOW | HIGH | LOW | LOW | LOW |
| Monma (2024) | HIGH | HIGH | HIGH | HIGH | LOW | LOW | HIGH | LOW | LOW | HIGH |
| Seng (2021) | HIGH | HIGH | HIGH | HIGH | LOW | HIGH | LOW | LOW | HIGH | LOW |
| Tholl (2024) | HIGH | HIGH | HIGH | LOW | LOW | HIGH | LOW | LOW | LOW | LOW |

LOW = low risk; MOD = moderate risk, HIGH = high risk

1. Was the study’s target population a close representation of the national population in relation to relevant variables, e.g. age, sex, occupation?
2. Was the sampling frame a true or close representation of the target population?
3. Was some form of random selection used to select the sample, OR, was a census undertaken?
4. Was the likelihood of non-response bias minimal?
5. Were data collected directly from the subjects (as opposed to a proxy)
6. Was an acceptable case definition used in the study?
7. Was the study instrument that measured the parameter of interest (e.g. prevalence of low back pain) shown to have reliability and validity (if necessary)?
8. Was the same mode of data collection used for all subjects?
9. Was the length of the shortest prevalence period for the parameter of interest appropriate?
10. Were the numerator(s) and denominator(s) for the parameter of interest appropriate?
11. Summary item on the overall risk of study bias
